# Metabolite Profiling, Modulation of Glucose Metabolism and Antioxidant Activity Reveals the Protective Effect of Sulfated Polysaccharides From *Gracilaria gracilis* in Hepatic Tissues of Type 2 Diabetic Rats

**DOI:** 10.1002/fsn3.72425

**Published:** 2026-10-24

**Authors:** Lethiwe Mpungose, Tosin A. Olasehinde, Ochuko L. Erukainure, Ajit Kumar, Md. Shahidul Islam, Ademola O. Olaniran

**Affiliations:** ^1^ Department of Microbiology, School of Life Sciences University of KwaZulu‐Natal Durban South Africa; ^2^ Nutrition and Toxicology Division, Food Technology Department Federal Institute of Industrial Research Oshodi Lagos Nigeria; ^3^ Department of Biochemistry, School of Life Sciences University of Kwazulu‐Natal Durban South Africa

**Keywords:** glucolipotoxicity, hepatopathy, oxidative stress, red seaweeds, sulfated polysaccharides, Type 2 diabetes

## Abstract

Diabetic hepatopathy is one of the complications of Type 2 diabetes (T2D), and it's a major cause of mortality and morbidities associated with the disease. Seaweeds are good sources of bioactive compounds of medicinal importance and have shown potential as an alternative therapy for the treatment of diabetes. This study assessed the protective effect of sulfated polysaccharides (SPs) from a red seaweed specie ( *Gracilaria gracilis* ) against diabetic hepatopathy and its potential biochemical mechanisms in rats with T2D. SPs from *G. gracilis* (150 and 300 mg/kg bodyweight [bw]) were given orally to two groups of T2D rats, respectively. The diabetic and normal control groups were given water, while metformin served as the standard. Hepatic activities of glucose metabolic enzymes and oxidative stress parameters were assessed in the liver. Hepatic metabolites in liver tissues from the different groups were analyzed using chromatographic techniques. Induction of T2D led to significant decrease in glutathione level, superoxide dismutase, catalase, glutathione peroxidase, glutathione reductase, glutathione S‐transferase activities, while an increase in fructose‐1.6‐biphosphatase, glucose 6‐phosphatase and glycogen phosphorylase activities were observed in hepatic tissues. Glucose, lipid and amino acid metabolic pathways, and their metabolites were also dysregulated on induction of T2D. Treatment with SPs led to the reversal of the glutathione and malondialdehyde levels, as well as antioxidant enzyme activities, Furthermore a significant improvement in glucose, lipid, and amino acid metabolisms were observed in hepatic tissues of SPs treated diabetic rats. Our findings showed that SPs from *G. gracilis* exhibited protective effects against diabetic hepatopathy in T2D by attenuating lipotoxicity, oxidative stress, with concomitant improved glucose, lipid and amino acid metabolisms.

## Graphical abstract

Sulfated polysaccharides (SPs) from the red seaweed *Gracilaria gracilis* , given orally at 150 and 300 mg/kg body weight, protected against diabetic hepatopathy in Type 2 diabetic rats. SPs restored hepatic glutathione and antioxidant enzyme activities, reversed lipid peroxidation, and improved hepatic glucose, lipid, and amino acid metabolism. These findings position *G. gracilis* SPs as a potential functional food ingredient for mitigating liver complications associated with Type 2 diabetes.

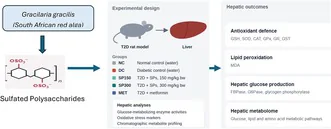

## 1 Introduction

Diabetes is a metabolic disorder characterized by hyperglycemia arising from the inability of the pancreatic β‐cells to secrete insulin or the inability of the body to utilize secreted insulin. The former is known as Type 1 diabetes (T1D), while the latter is Type 2 diabetes (T2D). Type 2 diabetes accounts for over 90% of all diabetes‐related morbidities and mortalities, thus making it the most common form of diabetes (IDF 2025). It is characterized by insulin resistance and pancreatic β‐cells, leading to hyperglycemia. Excessive hyperglycemia has been implicated in the production of reactive oxygen species (ROS) and free radicals, which leads to imbalance in the oxidant and antioxidant system. This imbalance is termed oxidative stress and is associated with the pathophysiology of T2D and its complications (Diyorbek 2025; Ren et al. 2025).

Diabetic hepatopathy is one of such complications associated with T2D arising from impaired glucose homeostasis in hepatic tissues (Erukainure et al. 2021). It is a non‐alcoholic fatty liver disease (NAFLD), and its clinical spectrum spans hepatosclerosis, glycogenic hepatopathy, abnormal liver enzyme profiles, cirrhosis, hepatitis C infection, and acute liver failure (Islam et al. 2020; Mobasheri et al. 2023; Tolman et al. 2007).

T2D‐induced insulin resistance alters hepatic glucose uptake and utilization, ultimately leading to exacerbated hepatic output and further contributing to blood glucose spikes (Erukainure et al. 2021). Alterations to lipid metabolic pathways and their associated metabolites also contribute to diabetic hepatopathy (Erukainure et al. 2021). The condition is reportedly caused by a heightened uptake of hepatic fatty acids, an increase in *de novo* lipogenesis, and a reduction in the secretion of very low‐density lipoprotein (VLDL), resulting in hypertriglyceridemia and hypercholesterolemia (Mobasheri et al. 2023; Westcott et al. 2024). Additionally, perturbation in amino acid metabolism exacerbates hepatic glucose production, which fuels the tissues' insensitivity to insulin, ultimately resulting in glucotoxicity (Ding et al. 2023; Zhu et al. 2022). The interplay of these pathophysiological activities results in glucolipotoxicity, with concomitant generation of ROS, ultimately leading to hepatic oxidative stress (Ma et al. 2021).

The role of sulfated polysaccharides (SPs) from seaweeds and their therapeutic effects against T2D and its complications have been investigated (Jia et al. 2020; Zhong et al. 2021). These polysaccharides can scavenge free radicals, inhibit carbohydrate digestive enzymes, improve hepatic lipid metabolism, hepatic functions and glucose metabolism, and promote muscle glucose uptake (Murakami et al. 2021; Pillay et al. 2025; Ponce and Stortz 2020; Wen et al. 2017; Zhang et al. 2022). *Gracilaria gracilis* is a red seaweed and belongs to the Gracilaria genus. The monosaccharide composition of its SPs has been reported to consist of glucose, mannuronic acid, galactose, arabinose, xylose, and glucuronic acid (Pillay et al. 2024). We previously reported that SPs from *G. gracilis* inhibit carbohydrate‐digestive enzyme activity and intestinal glucose absorption while promoting glucose uptake in isolated muscle in in vitro and ex vivo models (Pillay et al. 2024). The work of Makkar and Chakraborty (2017) also showed that SPs from *Gracilaria opuntia* possessed antidiabetic activity via the inhibitory effects on carbohydrate metabolizing enzymes (α‐amylase, α‐glucosidase, and dipeptidyl peptidase‐4). Also, Wen et al. (2017) reported that SPs from *Gracilaria lemaneiformis* exhibited antidiabetic effect in streptozotocin‐induced diabetic mice via reduction of blood glucose, repair of pancreatic cells, and regulation of lipid metabolism. Previous studies on the antidiabetic effects of SPs from some Gracilaria species have only focused on glucose and lipid regulation and oxidative stress. However, the effect of SPs from *G. gracilis* on diabetic hepatopathy has not been investigated. Therefore, the present study sought to investigate the effect of SPs obtained from *G. gracilis* on diabetic hepatopathy, by exploring its ability to improve hepatic metabolism of glucose, lipid, and amino acids as well as its ameliorative effect on oxidative stress in hepatic tissues of rats with T2D.

## 2 Materials and Methods

### 2.1 Sample Collection, Preparation and Characterization

*Gracilaria gracilis* was obtained from Wild Coast Abalone in the Eastern Cape Province of South Africa and identified as reported previously (Olasehinde et al. 2019). The seaweed was rinsed, freeze‐dried, and used to produce sulfated polysaccharides, as reported previously. The sulfated polysaccharides have also been characterized previously in our laboratory via the determination of their glucose content, sulphate content, and monosaccharide composition using Gas chromatography and liquid chromatography Mass spectrometric analysis (Pillay et al. 2024). The sulfated polysaccharides were stored in airtight vials at 4°C.

### 2.2 Animals

Fifty‐one male Sprague Dawley (SD) rats (180–200 g body weight) were obtained from the Biomedical Research Unit (BRU), University of KwaZulu‐Natal, Durban, South Africa. Animals were acclimatized for 7 days with free access to pelletized chow and water, under a natural 12‐h light–dark photoperiod. All experimental procedures received prior approval from the University of KwaZulu‐Natal Animal Ethics Committee (approval no. AREC/00002347/2021).

### 2.3 Animal Groupings

Following acclimatization, animals were assigned to six groups:

- Normal control (NC): non‐diabetic rats.
- Diabetic control (DC): untreated diabetic rats.
- Diabetic + metformin (DBM): diabetic rats given the standard drug metformin (200 mg/kg bw).
- Diabetic + low‐dose SPs (DRL): diabetic rats given *G. gracilis* SPs at 150 mg/kg bw.
- Diabetic + high‐dose SPs (DRH): diabetic rats given *G. gracilis* SPs at 300 mg/kg bw.
- Normal toxicological control (NTR): non‐diabetic rats given the high dose (300 mg/kg bw) of *G. gracilis* SPs.

The normal groups (NC and NTR) comprised 5 rats each, while the remaining four groups comprised 6 rats each. Dose selection was guided by previous reports. The selected doses were based on previous reports (Wen et al. 2017; Jia et al. 2020).

### 2.4 Induction of Type 2 Diabetes

T2D was induced in the diabetic groups using the fructose‐streptozotocin method (Wilson and Islam 2012). The DBC, DBM, DRL, and DRH groups received 10% fructose solution ad libitum for 2 weeks. Following an overnight fast, rats in these groups were given a single intraperitoneal injection of streptozotocin (40 mg/kg bw) dissolved in citrate buffer (pH 4.5), while the NC and NTR groups received citrate buffer alone by the same route. Non‐fasting blood glucose was measured 7 days later using an Accu‐Chek glucometer; animals with readings above 200 mg/dL were classified as diabetic.

### 2.5 Intervention Trial

Once T2D was confirmed, animals in the DRL, DRH, and NTR groups received their respective oral doses of *G. gracilis* SPs, the NC group received distilled water, and the DBM group received metformin. Treatments were administered 5 days per week across a 4‐week intervention period. Body weight and blood glucose were monitored weekly.

### 2.6 Sacrifice and Collection of Hepatic Tissue

Following an 8‐h overnight fast, rats were humanely euthanized using isoflurane. Blood was drawn by cardiac puncture into sterile plain tubes, after which the animals were dissected and their livers excised. Liver tissue was rinsed of blood with normal saline, blotted dry with filter paper, and weighed. A 0.5 g portion of each liver was homogenized in 5 mL of 50 mM sodium phosphate buffer (pH 7.5) containing 10% Triton X‐100. Homogenates were centrifuged for 10 min at 15,000 rpm and 4°C, and the resulting supernatants were stored in Eppendorf tubes at −80°C pending further analysis.

### 2.7 Assessment of Glucogenic Enzyme Activities

Hepatic activities of the glucogenic enzymes fructose‐1,6‐bisphosphatase, glucose‐6‐phosphatase, and glycogen phosphorylase were determined using previously described protocols (Balogun and Ashafa 2017; Cornblath et al. 1963; Erukainure et al. 2017; Gancedo and Gancedo 1971; Mahato et al. 2011).

#### 2.7.1 Fructose‐1,6‐Bisphosphatase Activity Assay

The tissue (100 μL) was added to a mixture of 100 μL of 0.05 M fructose, 1200 μL of 0.1 M Tris–HCl buffer (pH 7.0), 250 μL 0.1 M MgCl_2_, 100 μL 0.1 M KCl, and 250 μL 1 mM EDTA at 37°C and incubated for 15 min. TCA (10%) was added to the mixture to stop the reaction. The solution was spun for 10 min in a centrifuge set at 3000 rpm and 4°C. In a 96‐well plate, 50 μL of ascorbic acid (9%) and ammonium molybdate (1.25%) were mixed with 100 μL of the supernatant. After 20 min, the absorbance of the mixture was measured at 680 nm. The enzyme activity was extrapolated from an inorganic phosphate (Pi) standard graph.

#### 2.7.2 Evaluation of Glucose‐6‐Phosphatase Activity

A mixture of glucose‐6‐phosphatase (0.25 M, 100 μL), 200 μL of 5 mM KCl, and 1300 μL of 0.1 M Tris–HCl buffer was placed in a tube, and 200 μL of the tissue homogenate was added. The solution was placed in a shaker set at 37°C for 30 min. One milliliter of ammonium molybdate (1.25%) and distilled water were added to the solution to stop the reaction. Additionally, ascorbic acid (95, 1 mL), which was freshly prepared, was added to the solution. After 30 min. The absorbance of the solution was measured at 660 nm. The enzyme activity was extrapolated from an inorganic phosphate (Pi) standard graph.

#### 2.7.3 Assessment of Glycogen Phosphorylase Activity

The tissue (100 μL) was mixed with glycogen (4%) and glucose‐1‐phosphate (64 mM). The solution was incubated for 10 min at 30°C. A solution of ammonium molybdate (20%) in concentrated H_2_SO_4_ was added to the solution to stop the reaction. The solution was incubated with Elon reducer and distilled water at 30°C for 45 min. Absorbance was read at 340 nm. The enzyme activity was extrapolated from an inorganic phosphate (Pi) standard graph. The enzyme activity was extrapolated from an inorganic phosphate (Pi) standard graph.

### 2.8 Evaluation of Oxidative Stress Markers

Oxidative stress biomarkers such as reduced glutathione (GSH) level, superoxide dismutase (SOD), glutathione reductase (GR), glutathione peroxidase (GPx), glutathione‐S‐transferase (GST), catalase (CAT) activities, and lipid peroxidation were evaluated in liver tissues of the groups, according to previous methods (Aebi 1984; Bauman et al. 1988; Chowdhury and Soulsby 2002; Ellman 1959; Habig et al. 1974; Janero 1990; Jelodar et al. 2020).

#### 2.8.1 Evaluation of GSH Levels in Liver Tissues

Tissue homogenate (150 μL) was mixed with an equal volume of 10% TCA and centrifuged at 2000 rpm for 10 min at 25°C. The resulting supernatant (80 μL) was combined with 40 μL of 0.5 mM DTNB in a 96‐well plate and incubated with 200 μL of 0.2 M phosphate buffer (pH 7.8) at room temperature for 15 min. Absorbance was read at 415 nm, and GSH levels were extrapolated from a standard curve.

#### 2.8.2 Glutathione Reductase Activity

In a 96‐well plate, 221 μL of Tris–HCL (50 mM, pH 8.0) buffer with EDTA (1 mM), as well as 38 μL of oxidized glutathione (8 mM) were mixed with the tissue samples (10 μL). Ten microliters of NADPH were added to the plate, and the absorbance of the mixture was read at 340 at a 2 min interval for 8 min. Enzyme activity was obtained using the formula below:

$$\begin{array}{c} \text{Activity}=1000\times \left[\frac{A_{1}-A_{b}}{\varepsilon_{340}}\right]\times 0.5\;\mu \mathrm{mol}/\min/\mu g\;\text{of protein} \end{array}$$

where *ε*_340_ is the molar absorptivity at 340 nm, which is 6.22 mM/cm. *A*_1_ and *A*_b_ represent the rate of the sample and blank reactions.

#### 2.8.3 Estimation of Glutathione Peroxidase Activity

In a 96‐well plate, phosphate buffer (210 μL, pH 6.9), glutathione (100 mM, 2.5 μL), 5 μL of distilled water and 5 μL of the tissue homogenate were mixed. The solution was incubated at 37°C for 10 min, after which NADPH (2.5 μL, 15 mM) and DTNB (10 μL, 1 mM) were added. The absorbance of the solution was read at 412 nm. GPx concentration was extrapolated from a standard graph using GSH.

#### 2.8.4 Superoxide Dismutase Activity

A 0.1 mM diethylenetriaminepentaacetic acid (170 μL) was freshly prepared and mixed with 15 μL of liver tissue homogenate and 6‐HD (1.6 mM, 15 μL) in a 96‐well plate. The plate was gently tapped for proper mixing, and the absorbance was read at 492 nm at an interval of 1 min for 5 min. SOD activity was determined using the expression below:

$$\begin{array}{c} \text{Activity}=1000\times \left[\left(A_{1}-A_{b}\right)/\varepsilon_{490}\right]\times 0.5\;\text{nmol}/\min/\text{μ}\text{g}\;\text{protein} \end{array}$$

*ε*_490_ = Molar absorptivity at 490 nm = 1.742/mM/cm. *A*_1_ and *A*_*b*_ = reaction rate for sample and blank, respectively.

#### 2.8.5 Catalase Activity

Ten microliters of the tissue supernatant were mixed with 340 μL of 50 mM sodium phosphate buffer (pH 7.0). 150 μL of 2 M H_2_O_2_ was added to the reaction mixture. Absorbance was read at 240 nm for 3 min at 1 min interval.

#### 2.8.6 Glutathione‐S‐Transferase Activity

A reaction mixture of 0.1 mL of 0.25 M phosphate buffer (pH 6.5), 25 mM 1‐chloro‐2,4‐dinitrobenzene (CDNB), and 0.7 mL of distilled water was incubated for 5 min at 37°C. 25 μL of the tissue supernatants and 20 μL of 25 mM glutathione were added to the solution. The absorbance was read at 340 nm at intervals for 5 min.

#### 2.8.7 Lipid Peroxidation Level

Lipid peroxidation levels of the tissues were determined by measuring thiobarbituric acid reactive substances (TBARS), expressed as malondialdehyde (MDA) equivalent. 100 μL of the samples were mixed with an equal volume of 8.1% SDS solution, 375 μL of 20% acetic acid, 1 mL of 0.25% thiobarbituric acid (TBA), and 425 μL of distilled water. The solution was placed in a boiling bath. After 1 h, the absorbance of the solution was read at 532 nm. TBARS concentration was determined using a standard curve.

### 2.9 Hepatic Function Markers

The collected blood samples were centrifuged at 10,000 rpm at 4°C for 10 min. The supernatant (serum) was collected into 2 mL Eppendorf tubes and analyzed for serum hepatic function markers which include aspartate transaminase (AST) and alanine transaminase (ALT), using an Automated Chemistry Analyzer following the manufacturer's protocol (Labmax Plenno, Labtest Inc., Costa Brava, Brazil).

### 2.10 Metabolic Profiling

About 0.5 g of the liver was excised and homogenized in cold methanol/ethanol (2:8) to extract the lipid metabolites (Erukainure et al. 2020). The mixture was placed on ice as previously reported (Chan et al. 2013), and after 20 min, it was centrifuged at 15,000 rpm at 4°C for 10 min. The supernatants were decanted into glass vials for subsequent analysis.

#### 2.10.1 Gas Chromatography–Mass Spectrometry (GC–MS) Analysis of Hepatic Metabolites

The extracted metabolites were profiled with GC–MS using an Agilent Technologies 6890 Series GC coupled with an Agilent 5973 Mass Selective detector and driven by Agilent chemstation software. The metabolites were separated with a HP‐5MS capillary column. 1 μL of the tissue supernatants were injected in a spitless mode. Carrier gas: ultra‐pure helium used as the carrier gas; Flow rate: 60 mL h^−1^; Initial oven temperature: 60°C for 2 min; Final oven temperature: 285°C at the rate of 5°C min^−1^; Hold time: 3 min; Ion source and quadrupole Temperatures: 230°C and 150°C, respectively; Electron ionization mode and electron multiplier voltage: 70 eV and 1859 V, respectively. The metabolites were identified by direct mass spectral comparison with NIST mass spectral library.

#### 2.10.2 Pathway Analysis

The identified metabolites were subjected to pathway enrichment to identify the most relevant pathways involved in lipid metabolism in diabetic hepatopathy in T2D, using the MetaboAnalyst 6.0 online server (https://www.metaboanalyst.ca/) (Pang et al. 2024). Following metabolite upload, MetaboAnalyst 6.0 automatically matched the compounds and performed over‐representation analysis against SMPDB pathway‐associated metabolite sets using the hypergeometric test. No formal significance threshold was applied. Pathways were ranked using the enrichment *p*‐values automatically generated by MetaboAnalyst and were interpreted as exploratory pathway association.

### 2.11 Statistical Analysis

Clustering analysis of the GC–MS identified metabolites which covers for heat maps and principal component analysis (PCA) was carried out with MetaboAnalyst 6.0 online server (https://www.metaboanalyst.ca/) (Pang et al. 2024). Data were subjected to one‐way analysis of variance (ANOVA) and presented mean ± SD (standard deviation). Significant differences between means were set at *p <* 0.05. The Tukey's HSD multiple range post hoc test was used for multiple‐comparison.

## 3 Results

Hepatic activities of fructose‐1,6‐biphosphatase, glucose 6‐phosphatase, and glycogen phosphorylase were significantly (*p* < 0.05) increased in the diabetic control group (DBC) as shown in Figure 1A–C. Treatment with SPs led to a significant (*p* < 0.05) decrease in fructose‐1,6‐biphosphatase, glucose 6‐phosphatase, and glycogen phosphorylase activities in DRL, DRH, and DBM groups. In Figure 2A,B, significant (*p* < 0.05) depletion in hepatic activities of SOD and catalase was observed in the DBC group. These activities were significantly (*p* < 0.05) elevated following treatment with SPs and compared favorably with NC. There was a significant (*p* < 0.05) depletion in GSH level, and a decrease in glutathione reductase (GR), glutathione peroxidase (GPx), and glutathione S‐transferase (GST) activities in the hepatic tissue of rats in the DBC group as shown in Figure 3A–D. However, GSH levels, as well as GR, GST, and GPx activities, were significantly (*p* < 0.05) elevated following treatment with SPs in hepatic tissues of rats in DRL and DRH. There was a significant (*p* < 0.05) elevation in hepatic MDA level in hepatic tissues of rats in the DBC group as presented in Figure 4. The SPs significantly (*p* < 0.05) reduced MDA levels in hepatic tissues of rats in DRL and DRH groups.

**FIGURE 1:**
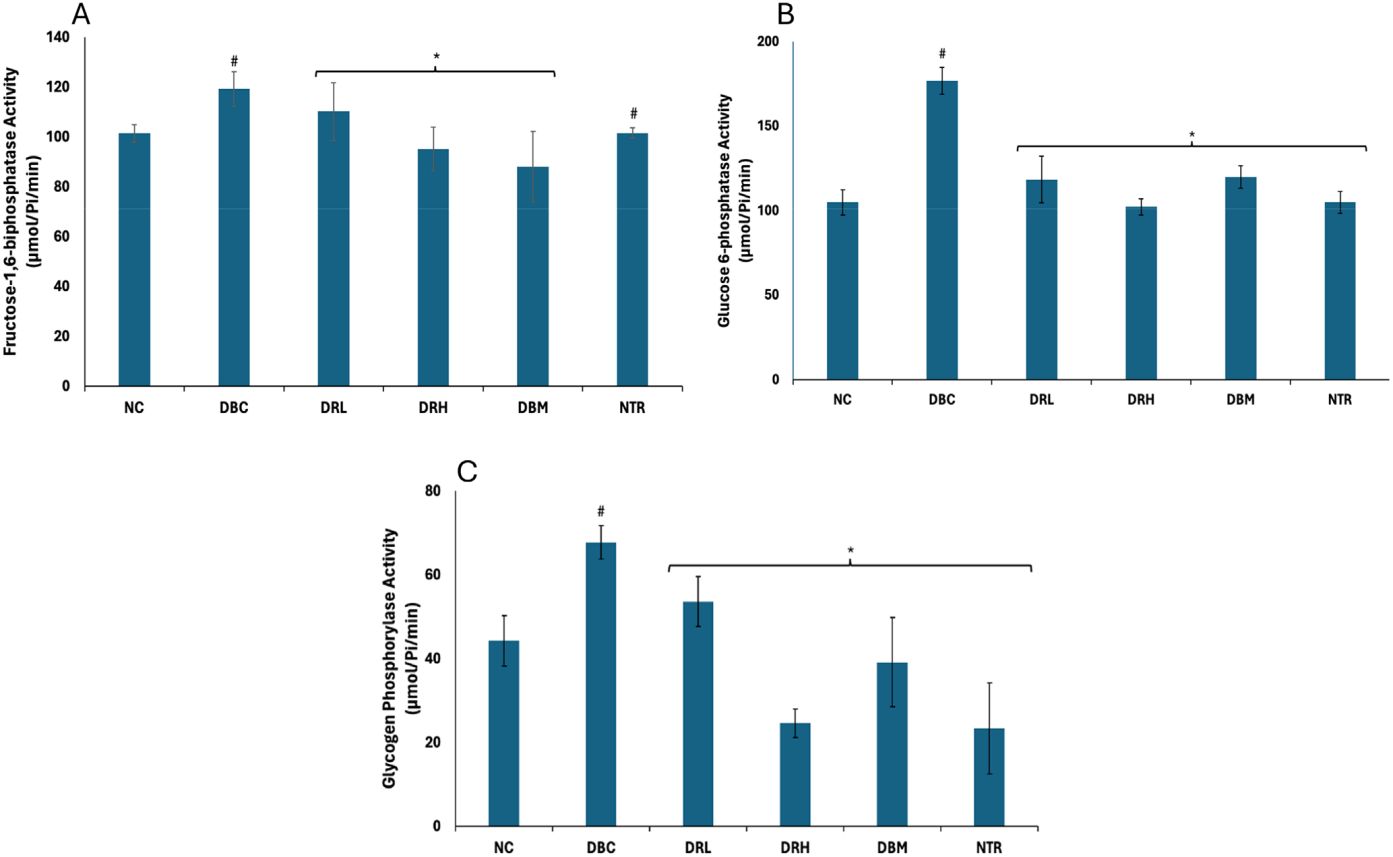
Effect of *Gracilaria gracilis* SPs on (A) fructose‐1,6‐biphosphatase, (B) glucose 6‐phosphatase, and (C) glycogen phosphorylase activities in hepatic tissues of T2D rats. Values = mean ± SD; *n* = 6. *Statistically significant (*p* < 0.05) to DBC; ^#^statistically (*p* < 0.05) significant to NC. DBC, diabetic control; DRH, diabetic + *G. gracilis* SP (300 mg/kg); DRL, diabetic + *G. gracilis* SP (150 mg/kg); NC, normal control; NTR, normal rats + *G gracilis* SP.

**FIGURE 2:**
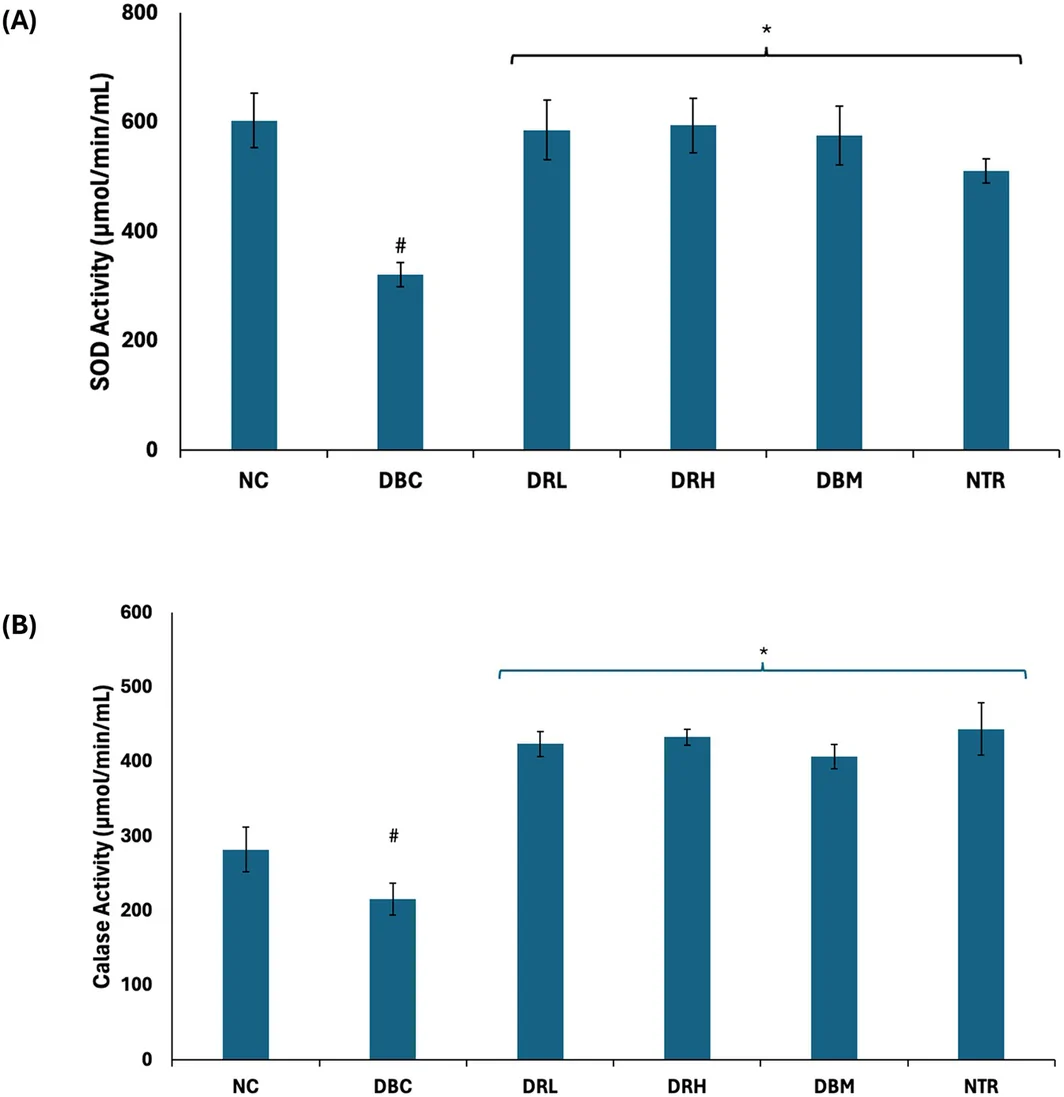
Effect of *Gracilaria gracilis* SPs on (A) SOD, and (B) catalase activities in hepatic tissues of T2D rats. Values = mean ± SD; *n* = 6. *Statistically significant (*p* < 0.05) to DBC; ^#^statistically (*p* < 0.05) significant to NC. DBC, diabetic control; DRH, diabetic + *G. gracilis* SP (300 mg/kg); DRL, diabetic + *G gracilis* SP (150 mg/kg); NC, normal control; NTR, normal rats + *G gracilis* SP (300 mg/kg).

**FIGURE 3:**
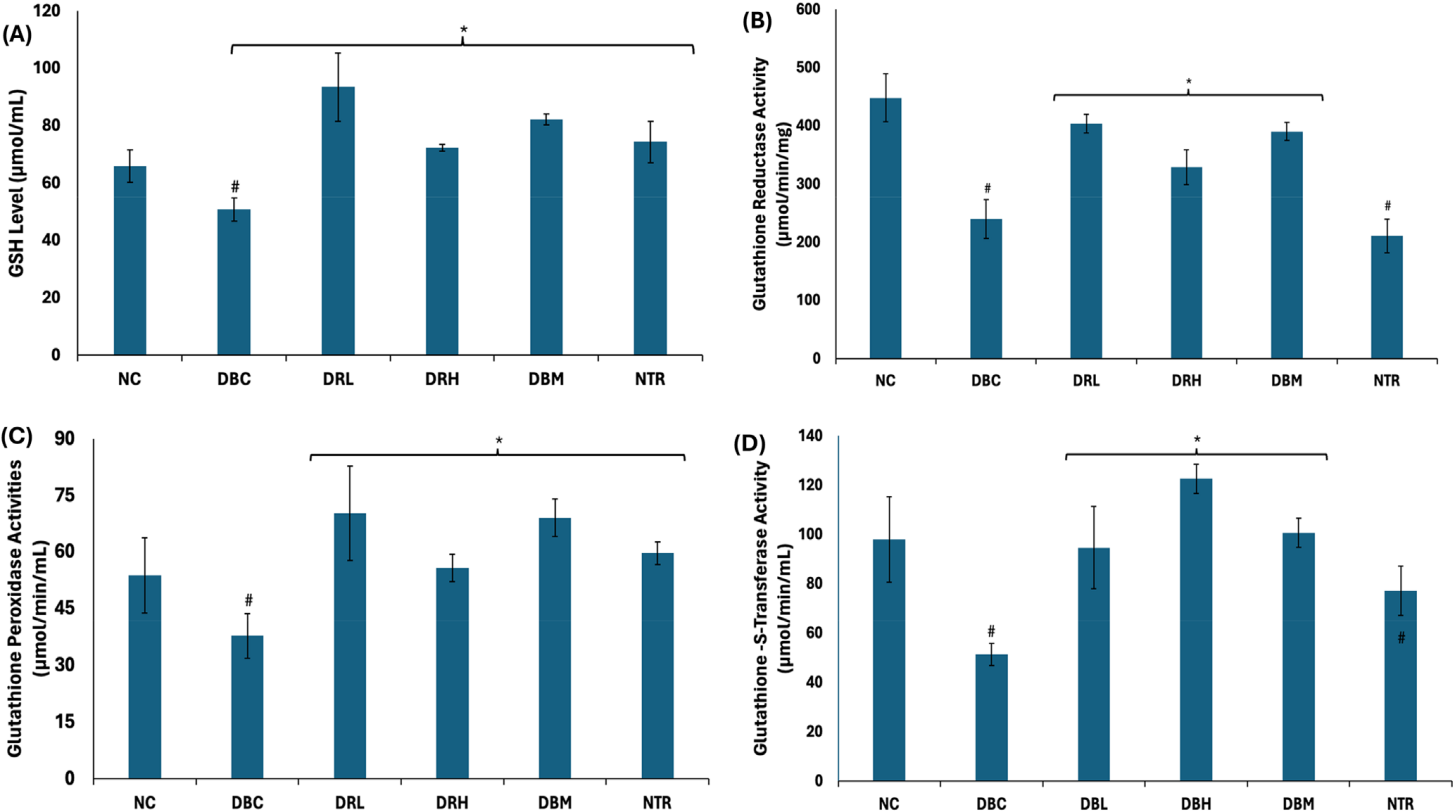
Effect of *Gracilaria gracilis* SPs on (A) GSH level, (B) glutathione reductase, (C) glutathione peroxidase, and (D) glutathione‐s‐transferase activities in hepatic tissues of T2D rats. Values = mean ± SD; *n* = 6. *Statistically significant (*p* < 0.05) to DBC; ^#^statistically (*p* < 0.05) significant to NC.

**FIGURE 4:**
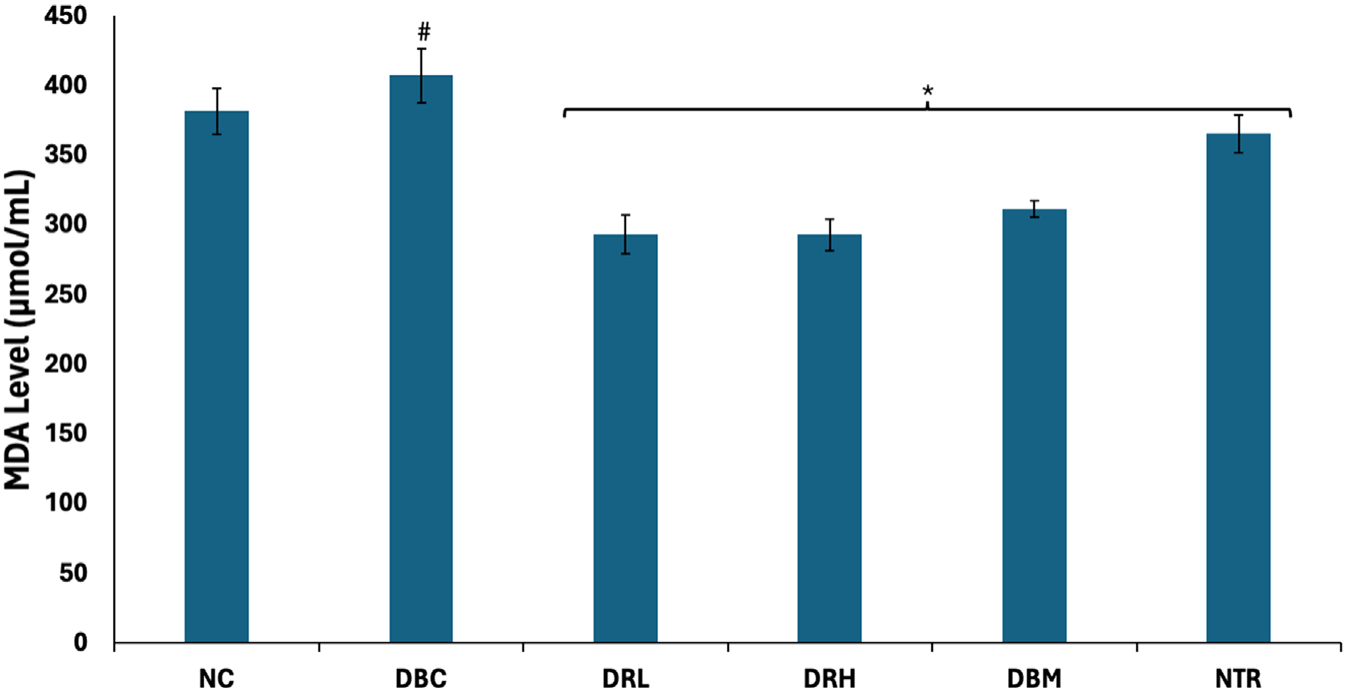
Effect of *Gracilaria gracilis* SPs on MDA level in hepatic tissues of T2D rats. Values = mean ± SD; *n* = 6. *Statistically significant (*p* < 0.05) to DBC; ^#^statistically (*p* < 0.05) significant to NC. DBC, diabetic control; DRH, diabetic + *G. gracilis* SP (300 mg/kg); DRL, diabetic + *G gracilis* SP (150 mg/kg); NC, normal control; NTR: normal rats + *G gracilis* SP (300 mg/kg).

AST and ALT activities were significantly (*p* < 0.05) elevated in serum of rats in the DBC group (Figure 5). The SPs significantly (*p* < 0.05) suppressed levels of the hepatic markers, except for AST and ALT levels in the DRL and DRH groups, respectively.

**FIGURE 5:**
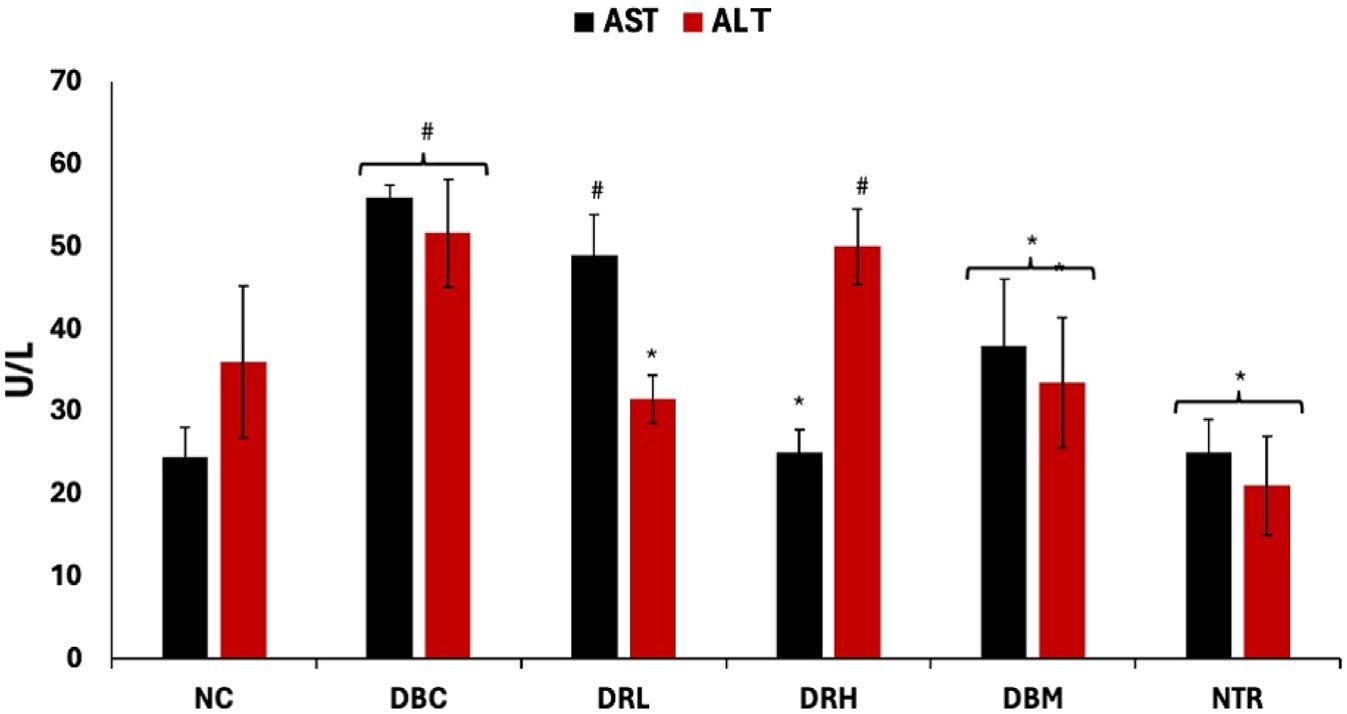
Effect of *Gracilaria gracilis* SPs on hepatic function markers levels in serum of T2D rats. Values = mean ± SD; *n* = 6. *Statistically significant (*p* < 0.05) to DBC; ^#^statistically (*p* < 0.05) significant to NC. DBC, diabetic control; DRH, diabetic + *G. gracilis* SP (300 mg/kg); DRL, diabetic + *G. gracilis* SP (150 mg/kg); NC, normal control; NTR, normal rats + *G gracilis* SP (300 mg/kg).

Hepatic metabolites present in NC group consist of urea, alpha‐ketoglutaric acid, palmitoleic acid, pentadecanoic acid, linoleic acid, (9e)‐9‐octadecenoic acid, cis‐vaccenic acid, octadecanoic acid, arachidonic acid, 12‐ketotridecanoic acid, eicosapentaenoic acid, cholesteryl nonanoate, cholesterol and dihydrobrassicasterol (Table 1). These metabolites were altered in DBC group as depicted by the depleted levels of arachidonic acid, dihydrobrassicasterol, eicosapentaenoic acid, and cis‐vaccenic acid. This was further depicted by the concomitant generation of nicotinamide, cholestadiene, 2‐hexyloctanoic acid, linolenic acid, 24‐dehydrocholesterol, heptadecanoic acid, squalene, and docosahexanoic acid. Treatment with SPs at low dose (DRL [150 mg/kg]) restored the eicosapentaenoic acid level, while depleting T2D‐generated nicotinamide, heptadecanoic acid, linolenic acid, docosahexanoic acid, and 24‐dehydrocholesterol. At high dose (DRH [300 mg/kg]), SPs depleted hepatic levels of pentadecanoic acid, linoleic acid, (9e)‐9‐octadecenoic acid, cis‐vaccenic acid, 12‐ketotridecanoic acid, and cholesteryl nonanoate. It also led to the depletion of T2D‐generated nicotinamide, heptadecanoic acid, and 24‐dehydrocholesterol, while concomitantly generating glycerin, 3‐methyldecanoic acid, pentadecanoic acid, cis‐10‐heptadecenoic acid, tetradecanoic acid, eicosanoic acid, and 4,5‐diamino‐6‐hydroxypyrimidine. Heatmap analysis of hepatic metabolite distribution among the treatment groups is presented in Figure 6A, with the DRL and DBC group portraying the most distinct changes as depicted by the positive value and color intensity. This was further portrayed by principal cluster analysis (PCA), which also depicted distinct changes in hepatic metabolites distribution between the SPs treated groups (DRL and DRRH) and untreated diabetic group (DBC) as shown by the score plots in Figure 6B. Biplot analysis of PCA components, PC1 (37.74%) and PC2 (24.76%) revealed strong relationships between treatment groups (DRL and DRH) and the hepatic metabolites: 4,5‐diamino‐6‐hydroxypyrimidine, eicosenoic acid, pentadecanoic acid, urea, linolenic acid, docosahexanoic acid, arachidonic acid, tetradecanoic acid, and 2‐hexyloctanoic acid (Figure 6C).

**TABLE 1:** Hepatic metabolites in percentage identified in control, untreated and treated T2D rats.

Metabolites	NC	DBC	DRL	DRH	DBM	NTR
Urea	0.47 ± 0.34	0.62 ± 0.29	0.75 ± 0.37	ND	ND	ND
Alpha‐Ketoglutaric acid	0.65 ± 0.45	1.40 ± 0.79	0.42 ± 0.18	1.01 ± 0.73	1.01 ± 0.64	1.35 ± 1.05
Palmitoleic acid	1.38 ± 0.17	1.53 ± 0.25	1.23 ± 0.43	1.43 ± 0.14	1.82 ± 0.68	0.99 ± 0.16
Pentadecanoic acid	12.14 ± 4.12	8.02 ± 1.81	5.40 ± 1.12	ND	8.18 ± 1.02	0.20 ± 0.10
Linoleic acid	1.53 ± 0.19	3.91 ± 1.08	1.15 ± 0.22	ND	5.19 ± 1.14	4.59 ± 1.47
(9E)‐9‐Octadecenoic acid	6.44 ± 0.30	3.43 ± 0.41	1.41 ± 0.12	ND	3.90 ± 0.38	ND
cis‐Vaccenic acid	3.99 ± 1.27	ND	ND	ND	ND	ND
Octadecanoic acid	4.61 ± 0.05	4.21 ± 0.37	5.01 ± 0.49	10.06 ± 3.00	5.60 ± 2.41	8.01 ± 1.81
Arachidonic acid	0.87 ± 0.38	ND	1.37 ± 0.41	0.95 ± 0.43	4.01 ± 0.07	5.48 ± 1.22
12‐Ketotridecanoic acid	0.68 ± 0.08	0.75 ± 0.12	1.05 ± 0.38	ND	ND	1.18 ± 0.24
Eicosapentaenoic acid	0.98 ± 0.08	ND	0.84 ± 0.17	ND	ND	0.87 ± 0.04
Cholesteryl nonanoate	0.74 ± 0.19	0.28 ± 0.10	ND	ND	ND	0.41 ± 0.06
Cholesterol	18.99 ± 4.38	23.99 ± 6.06	21.12 ± 8.56	12.54 ± 1.50	20.80 ± 4.05	14.42 ± 2.19
Dihydrobrassicasterol	2.04 ± 0.36	ND	ND	ND	ND	ND
2‐Hexyloctanoic acid	ND	2.99 ± 0.18	1.92 ± 0.19	0.29 ± 0.13	ND	2.11 ± 0.53
Nicotinamide	ND	0.51 ± 0.08	ND	ND	ND	ND
Heptadecanoic acid	ND	0.29 ± 0.12	ND	ND	ND	0.22 ± 0.06
Linolenic acid	ND	0.57 ± 0.10	ND	0.92 ± 0.28	ND	ND
Docosahexanoic acid	ND	0.66 ± 0.08	ND	1.13 ± 0.16	ND	0.28 ± 0.08
Squalene	ND	3.19 ± 1.25	1.60 ± 0.73	1.32 ± 0.58	ND	ND
Cholestadiene	ND	0.88 ± 0.38	0.50 ± 0.17	0.74 ± 0.10	0.96 ± 0.02	0.70 ± 0.03
24‐Dehydrocholesterol	ND	0.23 ± 0.01	ND	ND	ND	ND
17α‐Hydroxypregnenolone	ND	ND	4.53 ± 0.92	ND	ND	ND
Glycerin	ND	ND	ND	0.59 ± 0.30	ND	ND
3‐Methyldecanoic acid	ND	ND	ND	0.18 ± 0.09	ND	ND
Pentadecanoic acid	ND	ND	ND	0.37 ± 0.20	ND	ND
cis‐10‐Heptadecenoic acid	ND	ND	ND	0.23 ± 0.10	ND	ND
Tetradecanoic acid	ND	ND	ND	0.41 ± 0.19	ND	0.25 ± 0.13
Eicosanoic acid	ND	ND	ND	0.36 ± 0.13	ND	ND
4,5‐Diamino‐6‐hydroxypyrimidine	ND	ND	ND	0.41 ± 0.23	ND	0.27 ± 0.15
4‐Amino‐3‐isothiazolecarboxylic acid	ND	ND	ND	ND	ND	0.26 ± 0.14
Eicosenoic acid	ND	ND	ND	ND	ND	5.14 ± 1.69

*Note:* Values = mean ± SD; *n* = 6.

Abbreviation: ND, not detected.

**FIGURE 6:**
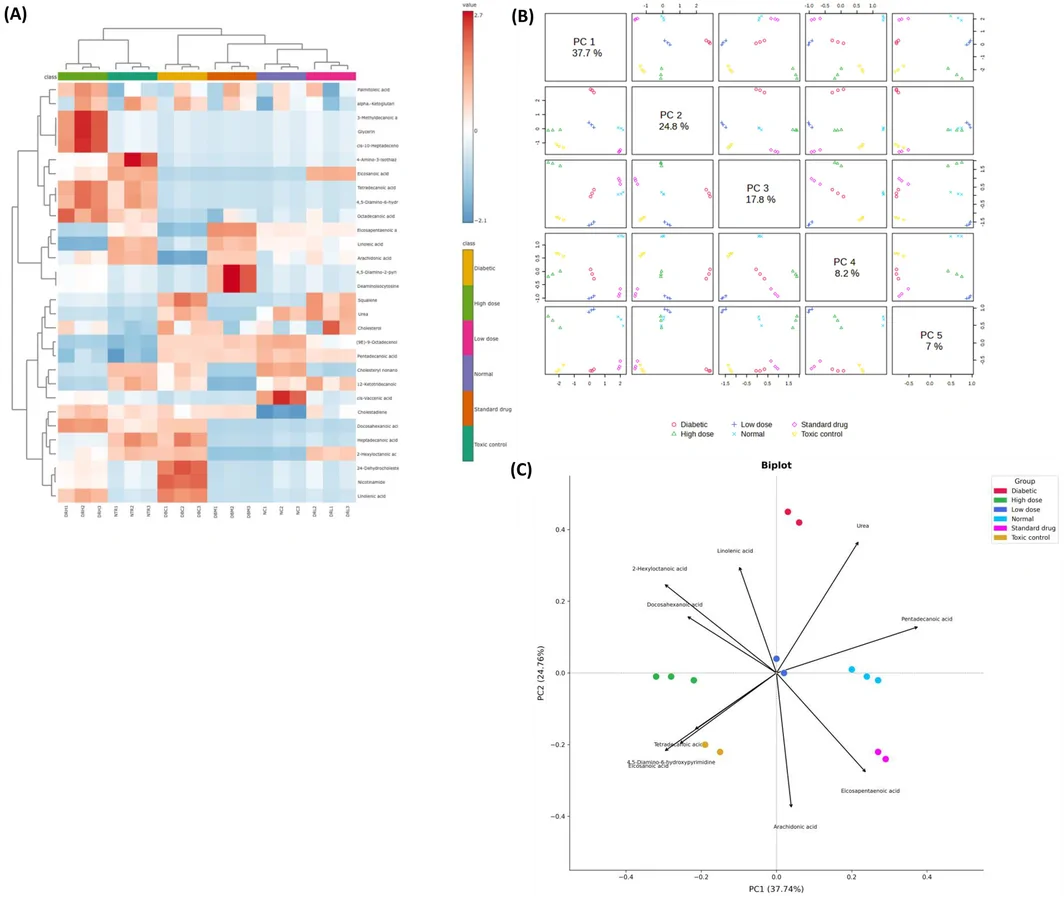
(A) Heat map; (B) PC scores; and (C) biplot relationship between selected PCs of hepatic metabolites in hepatic tissues of T2D rats. DBC, diabetic control; DRH, diabetic + *G. gracilis* SP (300 mg/kg); DRL, diabetic + *Gracilaria gracilis* SP (150 mg/kg); NC, normal control; NTR, normal rats + *G gracilis* SP (300 mg/kg).

From the pathway enrichment outcome of metabolites observed in hepatic tissues, our findings showed impaired regulation of glucose metabolism depicted by downregulated pathways for the citric acid cycle, malate–aspartate shuttle, and Warburg effect (Figure 7A and Table S1). These pathways were upregulated following treatment with both doses of SPs. At high dose, treatment with SPs triggered the activation of galactose metabolism. Induction of T2D also led to dysregulated lipid metabolism as shown in Figure 7B and Table S1. This is depicted by downregulation of linoleic and alpha‐linolenic acid metabolism, oxidation of branched‐chain fatty acids, phytanic acid peroxisomal oxidation, plasmalogen synthesis, mitochondrial beta‐oxidation of long‐chain saturated fatty acids, steroidogenesis, carnitine synthesis, and bile acid biosynthesis, while activating propanoate metabolism. Following treatment with SPs, the pathways were upregulated, while concomitantly inactivating propanoate metabolism. Fatty acid biosynthesis and glycerolipid metabolism were activated following treatment with SPs at high dose.

**FIGURE 7:**
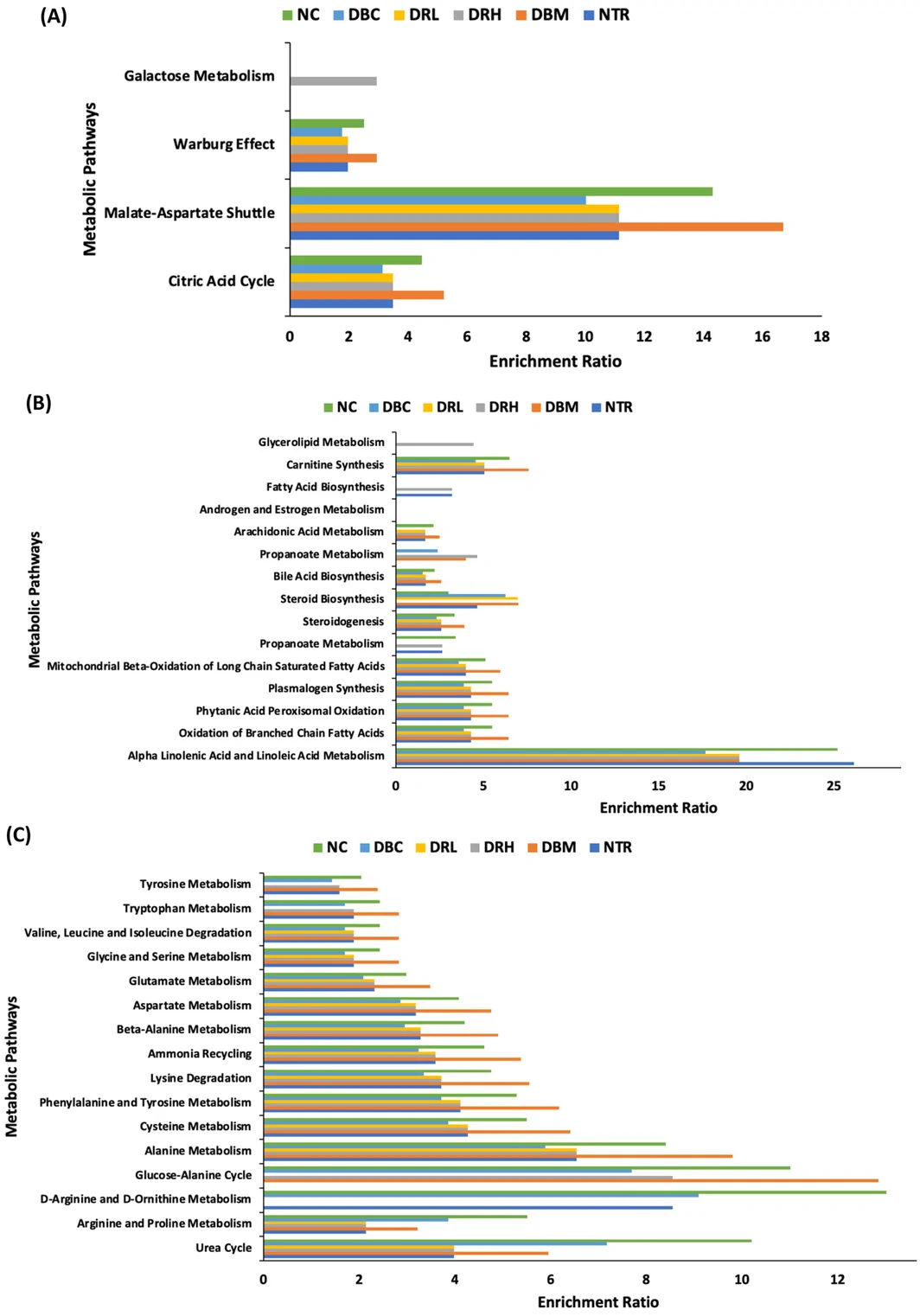
Pathway enrichment of hepatic metabolites for (A) glucose, (B) amino acids, and (C) lipid metabolism in hepatic tissues of T2D rats. DBC, diabetic control; DRH, diabetic + *G. gracilis* SP (300 mg/kg); DRL, diabetic + *Gracilaria gracilis* SP (150 mg/kg); NC, normal control; NTR, normal rats + *G. gracilis* SP (300 mg/kg).

Furthermore, induction of T2D led to dysregulated amino acid metabolism depicted by downregulation of the identified pathways (Figure 7C and Table S1). Treatment with SPs at low dose inactivated d‐arginine and d‐ornithine metabolism, glucose‐alanine cycle, tryptophan metabolism, and tyrosine metabolism, but upregulated the other pathways. The pathways, except d‐arginine and d‐ornithine metabolism, were upregulated following treatment with SPs at high dose.

## 4 Discussion

The increasing prevalence of diabetic hepatopathy is a major challenge and burden, as it is among the main contributors to T2D mediated mortality (Islam et al. 2020; Mallet et al. 2022; Mobasheri et al. 2023; Tolman et al. 2007). This has raised a need to explore effective and affordable therapy, with a paradigm shift toward natural bioactive ingredients. Among these naturally occurring products are seaweeds sulfated polysaccharides. In our previous study we reported the physicochemical properties of SPs from *G. gracilis* . The crude SP extract had an average molecular weight of 257.60 kDa, as determined by gel permeation chromatography. The sulfate content of the crude SPs was found to be 17.98% indicating the presence of sulfate groups in the polysaccharide structure. Some monosaccharides including glucose (42.01%), galactose (25.02%), arabinose, xylose, and glucuronic acid were identified in the SPs. The total sugar content of the crude SPs was 64.63%. However, in the present study, we investigated the effect of SPs from *G. gracilis* on diabetic hepatopathy in male rats with T2D.

Excessive hepatic glucose is a well‐recognized driver of hyperglycemia in diabetic hepatopathy (Barroso et al. 2024; Zhang et al. 2019). Particularly, unchecked glycogen breakdown exacerbate this effect in T2D (Jiang et al. 2020; Rines et al. 2016). In this study, we observed a marked increase in fructose‐1,6‐bisphosphatase, glucose‐6‐phosphatase, and glycogen phosphorylase activities in the diabetic control (DBC) group which pointed to active gluconeogenesis and glycogenolysis following T2D induction. The first two enzymes govern key steps of gluconeogenesis—conversion of fructose‐1,6‐bisphosphate to fructose‐6‐phosphate, and the terminal dephosphorylation of glucose‐6‐phosphate to free glucose—while glycogen phosphorylase initiates glycogen breakdown into glucose‐1‐phosphate, which is subsequently funneled toward glucose‐6‐phosphate. Together, the elevated activity of these three enzymes signals a shift away from glycolysis and toward disordered glucose metabolic pathways, with downstream consequences for the citric acid cycle, the malate–aspartate shuttle, and the Warburg effect, ultimately curtailing hepatic ATP availability (Koliaki and Roden 2013; Schmid et al. 2011). Treatment with SPs reversed this pattern, suppressing the activities of these enzymes (fructose‐1,6‐bisphosphatase, glucose‐6‐phosphatase, and glycogen phosphorylase) and upregulating glucose metabolic pathways, thereby inhibiting gluconeogenesis and glycogenolysis and improving production of ATP in the liver. This mirrors earlier work identifying suppression of gluconeogenesis and glycogenolysis as a viable strategy for managing diabetic hepatopathy (Rines et al. 2016). These findings are further supported by the study of Zheng et al. (2023), which revealed that *Fucus vesiculosus* ‐derived polysaccharides inhibited gluconeogenesis, promoted hepatic glycogen synthesis, and improved glycolipid metabolism in high‐fat diet and streptozotocin‐induced Type 2 diabetic rats. Together, these findings reveal the potential of SPs in improving hepatic glucose homeostasis in T2D, which is of significant translational relevance given that targeting excessive hepatic glucose production is a key therapeutic strategy for managing hyperglycemia and preventing the progression of diabetic hepatopathy. Persistent gluconeogenesis and glycogenolysis in T2D sustain elevated intracellular glucose, which is diverted into alternative pathways that generate toxic byproducts. Non‐enzymatic enolization of excess glucose can generate superoxide anion (O_2_^·−^) and reactive ketoaldehyde species, thereby disrupting the redox balance of the organ causing glucose‐induced oxidative damage (Snezhkina et al. 2019). Superoxide dismutase (SOD) converts O_2_^·−^ to hydrogen peroxide (H_2_O_2_), which degrades further into hydroxyl radicals (OH^·^) in cases where the peroxide is not reduced by glutathione peroxidase and catalase to water and oxygen (Meyerstein 2021). This was evident in this study by the observed decrease in hepatic activities of SOD and catalase in the DBC group. These reduced enzyme activities indicate hepatic oxidative stress in the diabetic rats. This corroborates previous studies linking oxidative stress with the pathophysiology of diabetic hepatopathy (Caturano et al. 2023; Oguntibeju 2019). Therefore, the elevated activities of these enzymes, following treatment with SPs, suggest mitigation of oxidative stress and antioxidant activity, which is a promising finding for the potential treatment of diabetic hepatopathy (Mobasheri et al. 2023; Mohamed et al. 2009). The study of Wen et al. (2017) also revealed that sulfated polysaccharides from *G. lemaneiformis* improved antioxidant enzymes in diabetic mice. *Laminaria japonica* polysaccharide also mitigated oxidative stress in the liver of streptozotocin‐induced diabetic rats (Xue et al. 2025). Oxidative stress plays a central role in the pathogenesis of liver damage in diabetic conditions, and targeting this process could help prevent or delay the progression of the disease. Antioxidant‐rich SPs from seaweeds may play an important therapeutic strategy in mitigating diabetic hepatopathy.

Excess glucose can be shunted to the polyol pathway, where it is converted into sorbitol by aldose reductase. Sorbitol is subsequently converted to fructose by sorbitol dehydrogenase (Erukainure and Chukwuma 2024). The first step of this pathway consumes NADH rather than allowing its oxidation to NAD^+^, which perturbs glutathione metabolism and promotes oxidative stress (Brownlee 2005). This is consistent with the reduced hepatic GSH levels in diabetic control rats (DBC). Also, reduced hepatic activities of glutathione reductase, glutathione peroxidase, and glutathione S‐transferase (GST) were observed in the diabetic rats. Oxidized glutathione (GSSG) is normally reduced to GSH in a reaction catalyzed by glutathione reductase, whereas glutathione peroxidase catalyzes the oxidation of GSH to GSSG (Couto et al. 2016; Erukainure and Chukwuma 2024). These decreased enzyme activities portray perturbed GSH homeostasis, which also indicates the occurrence of hepatic oxidative stress. The decreased GST activity further indicates elevated ROS levels (Szeligowska et al. 2022; Zhang et al. 2021). The increased enzyme activities and GSH level in the SPs‐treated diabetic rats (DRL and DRH) reveal improved glutathione metabolism. This also corroborates the improved antioxidative activities exerted by the sulfated polysaccharides in hepatic tissues of diabetic rats.

When catalase and glutathione peroxidase activity are impaired, hydroxyl radicals produced from the degradation of H_2_O_2_ attack membrane lipids, triggering peroxidation that yields malondialdehyde (MDA) and 4‐hydroxy‐2‐nonenal (HNE) (Ighodaro and Akinloye 2018; Jena et al. 2023). The high levels of hepatic MDA observed in the diabetic control rats (DBC) indicate lipid peroxidation, which shows hepatic oxidative stress correlates with the perturbed antioxidant enzyme activities and glutathione metabolism. Lipid peroxidation is central to the pathology of diabetic hepatopathy, and it is a good treatment target in disease management (de Souza Bastos et al. 2016; Shabalala et al. 2022). Thus, the decreased MDA levels following treatment with SPs indicate an ameliorative effect on lipid peroxidation in the hepatic tissues. This also corroborates antioxidative activities of the SPs in diabetic hepatopathy.

Insulin resistance is one of the main trigger for disrupted hepatic amino acid metabolism in diabetic hepatopathy (Cuomo et al. 2022; Wiklund et al. 2016) and this disruption typically manifests as elevated circulating transaminases (Judi et al. 2010; Yazdi et al. 2019). This is evident in our results by the dysregulated amino acid metabolic pathways, and concomitant elevated serum levels of aspartate transaminase (AST) and alanine transaminase (ALT) in the diabetic control rats (DBC). These dysregulated pathways can be linked to depleted hepatic urea and alpha‐ketoglutaric acid levels, with AST and ALT activities playing influential roles. ALT converts alanine to pyruvate and glutamate by transferring an amino group from alanine to α‐ketoglutarate, a crucial step in amino acid metabolism (Reaction 1). The reversal of this reaction is regarded as the glucose‐alanine cycle, which drives gluconeogenesis. The increased serum level of ALT indicates depleted hepatic availability of the enzyme for transamination activity. This is further portrayed by the reduced hepatic level of α‐ketoglutarate (Table 1) and dysregulated glucose‐alanine cycle, alanine metabolism, beta‐alanine metabolism and glutamate metabolism pathways (Figure 7 and Table S1).

(1) Alanine+α−Ketoglutarate⇌Pyruvate+Glutamate

AST converts aspartate to glutamate and oxaloacetate by catalyzing the transfer of the amino group from aspartate to alpha‐ketoglutarate (Reaction 2).

(2) Aspartate+α−Ketoglutarate⟷Oxaloacetate+Glutamate

The generated glutamate from both reactions is converted to ammonia (Reaction 3), which goes into the urea cycle. This corresponds with the dysregulated urea cycle, ammonia recycling, aspartate and glutamate metabolism pathways (Figure 8).

(3) $$\text{Glutamate}+{\mathrm{NAD}}^{+}+H_{2}O\to \alpha -\text{Ketoglutarate}+{\mathrm{NH}}_{3}+\text{NADH}+H^{+}$$

**FIGURE 8:**
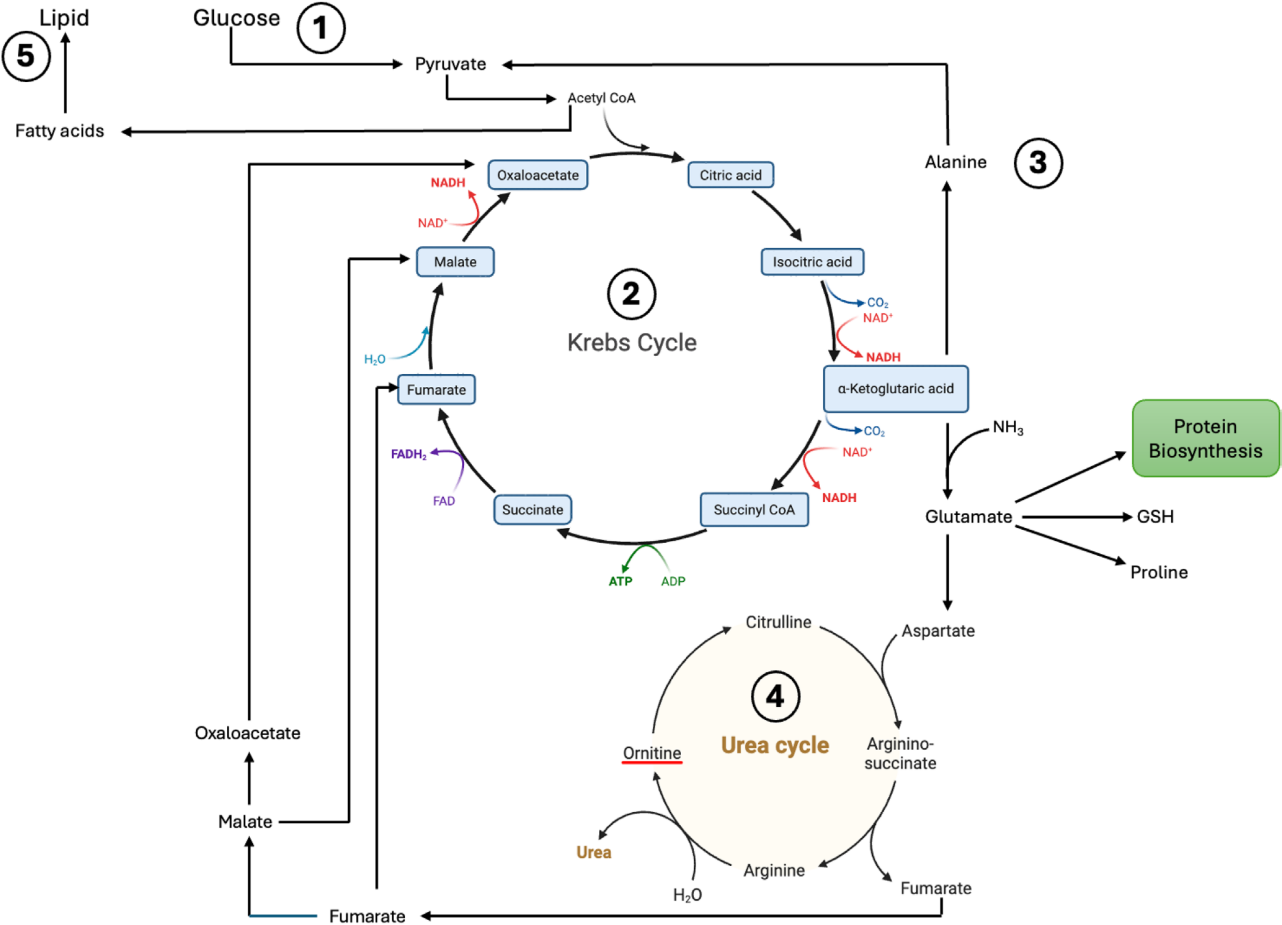
Schematic pathway by which SPs may improve protect against diabetic hepatopathy in T2D. (1) Glycolysis; (2) TCA/Krebs cycle; (3) glucose‐Alanine cycle; (4) urea cycle; and (5) de novo lipogenesis.

The upregulation of these pathways, alpha‐ketoglutarate concentration, and concomitant decreased serum levels of AST and ALT in the SPs‐treated diabetic rats (DRL and DRH) indicates improved hepatic amino acid metabolism. These findings correlate with the report of Xue et al. (2025) which revealed through metabolomics analysis that amino acid metabolism is a key pathway modulated by fucoidan treatment in the livers of diabetic rats, highlighting the importance of the amino acid metabolic pathway as a shared therapeutic target for seaweed‐derived SPs across different species. Hence the improvement of serum transaminase levels and amino acid metabolic pathways observed in the rat model corresponds directly to clinically measurable endpoints in diabetic hepatopathy, suggesting that these biomarkers could be valuable for monitoring therapeutic responses in future clinical studies.

Hepatic lipotoxicity which is caused by fat accumulation, impaired fatty acid oxidation, and altered steroid metabolism is a defining feature of diabetic hepatopathy (Gluchowski et al. 2017; Mendez‐Sanchez et al. 2018). Our results reflect this: diabetic control rats showed altered free fatty acid (FFA) profiles, including generation of additional FFA species and imbalance among existing ones (Table 1), consistent with hepatic fat accumulation and lipotoxicity following T2D induction. This finding aligns with disruption of hepatic lipid‐metabolic pathways, in particular branched‐chain fatty acid oxidation, peroxisomal oxidation of phytanic acid, and mitochondrial beta‐oxidation of long‐chain saturated fatty acids (Table S1). Concurrent disruption of steroidogenesis, steroid biosynthesis, and bile acid biosynthesis pathways may stem from altered hepatic cholesterol and related sterol metabolites. These observations are consistent with previous reports of disrupted lipid‐metabolic pathways and metabolite profiles in the livers of diabetic rats (Erukainure et al. 2021). Similarly, Zhang et al. (2024) reported that fucoidan from *L. japonica* reduced hepatic lipid accumulation and regulated lipid metabolism in diabetic rats via modulation of bile acids and gut microbiota, revealing the hepatoprotective potential of seaweed‐derived SPs. These findings align closely with those of Zheng et al. (2023), who reported that *F. vesiculosus* ‐derived polysaccharides significantly ameliorated dyslipidemia and liver dysfunction in Type 2 diabetic rats by inhibiting gluconeogenesis and regulating lipid metabolism‐related gene expression. Similarly, Zhang et al. (2024) demonstrated that *L. japonica* polysaccharide reduced hepatic fat accumulation and regulated lipid metabolism in diabetic rats through the modulation of bile acid secretion and gut microbiota composition. These findings from different seaweed‐derived SPs highlight a consistent hepatoprotective mechanism involving the regulation of lipid metabolic pathways, which may translate to clinical benefit in human patients with diabetic hepatopathy, where hepatic steatosis and lipotoxicity are major drivers of disease progression. The improved hepatic FFAs levels and constituents, with concomitant upregulation of lipid metabolic pathways following treatment with SPs in the diabetic rats (DRL and DRH) indicate suppressed hepatic lipid accumulation and improved metabolism. Thus, suggesting an anti‐lipotoxic effect on diabetic hepatopathy.

These findings lie in the potential of *G. gracilis* SPs as a novel therapeutic agent for the management of diabetic hepatopathy. The multi‐targeted action of these compounds on glucose, amino acid, and lipid metabolism, as well as their antioxidant effects, makes them a promising candidate for mitigating diabetic hepatopathy. However, while animal models of T2D and its complications share many features with human models, there are also significant differences in terms of genetics, physiology, and environmental factors that can influence the development and progression of the condition. Additionally, the dose and duration of SP treatment used in this study may not directly translate to human clinical settings due to factors such as bioavailability, safety, and tolerability. Hence, further preclinical studies that will optimize the dosing regimen, assess long‐term safety and efficacy, and elucidate molecular mechanisms underlying the observed metabolic and antioxidant effects are required. Additionally, clinical experiments involving the SPs in a diabetic hepatopathy model are also important to establish the translational relevance of these findings and determine their potential for clinical application.

## 5 Conclusion

This study revealed that SPs from *G. gracilis* ameliorated diabetic hepatopathy in a rat model of T2D. The SPs exerted a multi‐targeted hepatoprotective effect by suppressing gluconeogenesis and glycogenolysis, as evidenced by reduction in fructose‐1,6‐bisphosphatase, glucose 6‐phosphatase, and glycogen phosphorylase activities, coupled with the upregulation of key glucose metabolic pathways including the citric acid cycle, malate–aspartate shuttle, and Warburg effect. These results revealed improved hepatic glucose homeostasis and restoration of glucose oxidative metabolism in diabetic hepatic tissues. The SPs also demonstrated significant antioxidant activity in the hepatic tissues of diabetic rats, as reflected by the restoration of SOD and catalase activities, elevation of glutathione levels, and improvement in glutathione reductase, glutathione peroxidase, and glutathione S‐transferase activities, accompanied by a marked reduction in lipid peroxidation as indicated by decreased MDA levels. Furthermore, the SPs improved hepatic amino acid metabolism by restoring the activities of key transaminases, upregulating dysregulated amino acid metabolic pathways, and reducing elevated serum AST and ALT levels. The restoration of hepatic lipid metabolic pathways indicates that the SPs suppressed hepatic lipid accumulation and lipotoxicity in diabetic rats. Although these findings are promising, they are based on a rodent model. Future studies should focus on elucidating the molecular mechanisms underlying these effects, optimizing extraction and dosing strategies, assessing long‐term safety and bioavailability, and ultimately conducting well‐designed clinical trials to establish the translational relevance of these findings. The multi‐targeted effects of the *G. gracilis* SP make it a promising therapeutic strategy for the management of diabetic hepatopathy.

## Author Contributions

**Lethiwe Mpungose:** investigation, writing – original draft, methodology, visualization, software, formal analysis, data curation. **Tosin A. Olasehinde:** conceptualization, investigation, methodology, validation, visualization, writing – review and editing, software, formal analysis, project administration, data curation, supervision. **Ochuko L. Erukainure:** investigation, writing – original draft, methodology, visualization, software, formal analysis, data curation. **Ajit Kumar:** investigation, methodology, data curation. **Md. Shahidul Islam:** resources, writing – review and editing, validation, investigation. **Ademola O. Olaniran:** conceptualization, funding acquisition, writing – review and editing, supervision, project administration, resources, validation.

## Funding

This study was supported by a grant from Technology Innovation Agency (TIA), South Africa and South African Biodesign Initiative (Grant no. SABDI 16/1072), and Incentives for Rated Researchers by the National Research Foundation (NRF).

## Disclosure

All authors have read and approved the final version of the manuscript. Corresponding Author had full access to all of the data in this study and takes complete responsibility for the integrity of the data and the accuracy of the data analysis.

## Ethics Statement

This study was approved by the Animal Ethics Committee, University of KwaZulu‐Natal, Durban, South Africa (AREC/00002347/2021).

## Conflicts of Interest

The authors declare no conflicts of interest.

## Supporting information


**Table S1:** Pathway of identified hepatic metabolites of T2D rats.

## Data Availability

The data that support the findings of this study are available on request from the corresponding author. The data are not publicly available due to privacy or ethical restrictions.
