# Supplementary material for: Metabolite Profiling, Modulation of Glucose Metabolism and Antioxidant Activity Reveals the Protective Effect of Sulfated Polysaccharides From Gracilaria gracilis in Hepatic Tissues of Type 2 Diabetic Rats
Source: Food Sci Nutr. 2026 Oct 24;14(10):e72425. doi: 10.1002/fsn3.72425 (PMC13695034; doi:10.1002/fsn3.72425)
Supplement: Supplementary file 1 [file FSN3-14-e72425-s001.docx]

**Supplementary Table 1:** Pathway of identified hepatic metabolites of T2D rats.

| **Metabolism** | **Pathways** | **NC** | **DBC** | **DRL** | **DRH** | **DBM** | **NTR** |
| --- | --- | --- | --- | --- | --- | --- | --- |
| Glucose metabolism | Citric Acid Cycle |  |  |  |  |  |  |
|  | Malate-Aspartate Shuttle |  |  |  |  |  |  |
|  | Warburg Effect |  |  |  |  |  |  |
|  | Galactose Metabolism |  |  |  |  |  |  |
| Lipid Metabolism | Alpha Linolenic Acid and Linoleic Acid Metabolism |  |  |  |  |  |  |
|  | Oxidation of Branched Chain Fatty Acids |  |  |  |  |  |  |
|  | Phytanic Acid Peroxisomal Oxidation |  |  |  |  |  |  |
|  | Plasmalogen Synthesis |  |  |  |  |  |  |
|  | Mitochondrial Beta-Oxidation of Long Chain Saturated Fatty Acids |  |  |  |  |  |  |
|  | Steroidogenesis |  |  |  |  |  |  |
|  | Steroid Biosynthesis |  |  |  |  |  |  |
|  | Bile Acid Biosynthesis |  |  |  |  |  |  |
|  | Propanoate Metabolism |  |  |  |  |  |  |
|  | Arachidonic Acid Metabolism |  |  |  |  |  |  |
|  | Fatty Acid Biosynthesis |  |  |  |  |  |  |
|  | Carnitine Synthesis |  |  |  |  |  |  |
|  | Glycerolipid Metabolism |  |  |  |  |  |  |
| Amino acid metabolism | Urea Cycle |  |  |  |  |  |  |
|  | Arginine and Proline Metabolism |  |  |  |  |  |  |
|  | D-Arginine and D-Ornithine Metabolism |  |  |  |  |  |  |
|  | Glucose-Alanine Cycle |  |  |  |  |  |  |
|  | Alanine Metabolism |  |  |  |  |  |  |
|  | Cysteine Metabolism |  |  |  |  |  |  |
|  | Phenylalanine and Tyrosine Metabolism |  |  |  |  |  |  |
|  | Lysine Degradation |  |  |  |  |  |  |
|  | Ammonia Recycling |  |  |  |  |  |  |
|  | Beta-Alanine Metabolism |  |  |  |  |  |  |
|  | Aspartate Metabolism |  |  |  |  |  |  |
|  | Glutamate Metabolism |  |  |  |  |  |  |
|  | Glycine and Serine Metabolism |  |  |  |  |  |  |
|  | Valine, Leucine and Isoleucine Degradation |  |  |  |  |  |  |
|  | Tryptophan Metabolism |  |  |  |  |  |  |
|  | Tyrosine Metabolism |  |  |  |  |  |  |

= present; = downregulated; = upregulated; = inactivated; = no change
